# Epidemiology of growth hormone deficiency in children and adolescents: a systematic review

**DOI:** 10.1007/s12020-024-03778-4

**Published:** 2024-03-18

**Authors:** Chiara Mameli, Liliana Guadagni, Massimiliano Orso, Valeria Calcaterra, Malgorzata Gabriela Wasniewska, Tommaso Aversa, Simona Granato, Pietro Bruschini, Daniela d’Angela, Federico Spandonaro, Barbara Polistena, Gianvincenzo Zuccotti

**Affiliations:** 1 Department of Pediatrics, Buzzi Children’s Hospital, Milan, Italy; 2 Department of Biomedical and Clinical Science, Università Di Milano, Milan, Italy; 3 Department of Surgical and Biomedical Sciences, University of Perugia, Perugia, Italy; 4 C.R.E.A. Sanità (Centre for Applied Economic Research in Healthcare), Rome, Italy; 5 Department of Pediatrics, Ospedale dei Bambini V. Buzzi, Milan, Italy; 6 Department of Internal Medicine and Therapeutics Università degli Studi di Pavia, Pavia, Italy; 7 Pediatric Unit, AOU Policlinico “G. Martino”, Messina, Italy; 8 Department of Human Pathology of Adulthood and Childhood, University of Messina, Messina, Italy; 9 Medical Department, Pfizer Italia, Rome, Italy; 10 University of Rome Tor Vergata, Rome, Italy

**Keywords:** Growth hormone deficiency, GHD, Epidemiology, Prevalence, Incidence

## Abstract

**Objective:**

Growth hormone deficiency (GHD) is the most common pituitary hormone deficiency and is one of the main causes of short stature in children and adolescents. The aim of this study is to evaluate the epidemiology of pediatric GHD worldwide, since no other systematic review has been published so far.

**Methods:**

We searched PubMed, Embase, and Web of Science up to July 2023 to find epidemiological studies involving children with GHD. Two review authors independently screened articles, extracted data and performed the quality assessment.

**Results:**

We selected 9 epidemiological studies published from 1974 to 2022. The range of prevalence was 1/1107–1/8,646. A study based on a registry of GH users in the Piedmont region (Italy) reported the highest mean prevalence. In the included studies, the mean incidence ranged from 1/28,800 to 1/46,700 cases per year. One study reported a 20-year cumulative incidence of 127/100,000 for boys and 93/100,000 for girls. Studies were heterogeneous in terms of population (age and GHD etiology) and diagnostic criteria. As for the methodological quality of included studies, all but one study satisfied the majority of the checklist items.

**Conclusions:**

The included studies are mostly European, so the provided estimates cannot be considered global. International multicentre studies are needed to compare epidemiological estimates of GHD among different ethnical groups. Considering the considerable cost of human recombinant GH, the only available therapy to treat GHD, understanding accurate epidemiological estimates of GHD in each country is fundamental for resource allocation.

## Introduction

Growth Hormone (GH) or somatotropin is a 191-amino-acid peptide, synthesized and secreted by somatotrope cells in the anterior portion of the pituitary gland, that is mainly responsible for growth, cell reproduction ad regeneration studied in human and animal models [1].

Growth hormone deficiency (GHD) is an endocrine disorder, which may be classified according to the disease onset (pediatric or adult onset), the cause or mechanism (congenital, acquired or idiopathic), deficiency intensity, duration, and according to the involvement of other pituitary hormones or as part of a complex syndrome (isolated or part of Multiple Pituitary Hormone Deficiency) [2–4]. During pediatric age most cases are isolated and the majority of them are idiopathic.

The diagnosis of GHD is established on clinical, biochemical and radiological diagnostic criteria. Auxology, radiographic assessment of bone age, measurement of insulin-like growth factor 1 and IGF binding protein 3, GH provocative testing, cranial magnetic resonance imaging, and genetic testing (in some cases) are usually required to reach the diagnosis [5].

Recombinant human GH (rhGH) should be administered to patients affected by GHD as soon as the diagnosis is made to promote growth during childhood and achieve adequate adult height.

During the last 70 years, studies on the epidemiology of GHD have been published. They were mostly based on hospital records, institution-based registry as well as on national registries.

Currently, there is no published work that collects and analyses child-onset GHD data, so the aim of this systematic review is to evaluate the epidemiology of pediatric GHD and execute meta-analysis whenever possible.

## Material and methods

We performed the review in accordance with the PRISMA 2020 guidelines [6, 7]. The protocol of this systematic review was registered in PROSPERO (CRD42022350450).

We selected studies according to the following inclusion and exclusion criteria.

### Inclusion criteria

- P = children/adolescents (<18 years) with growth hormone deficiency (GHD)
- I = not applicable
- C = not applicable
- O = incidence, prevalence
- S = population-based cross-sectional studies, population-based cohort studies

### Exclusion criteria

- non-human studies;
- reviews, editorials, commentaries, letters.

### Information sources

Systematic searches were performed in PubMed, Embase and Web of Science from databases inception to July 2023. Reference lists of relevant articles were also screened. No date or language limits were imposed on the search.

### Search strategy

Literature search strategies were developed using medical subject headings (MeSH) and text words related to growth hormone deficiency and epidemiology. The full search strategy for the three databases is reported in the Supplementary Table 1.

### Selection process

The study selection process was performed by two independent review authors (MO, LG). Any disagreement was solved through discussion and, when necessary, a third reviewer was contacted (CM). Study selection was conducted in two phases. Initially, the reviewers assessed the records through the titles and abstracts screening against the inclusion criteria. In the second phase, the review authors assessed the full texts of the potential eligible studies. The final studies included in the review were described in the main text and in the tables, while a list of excluded studies along with the reasons for exclusion has been published as Supplementary Table 2.

### Data collection process

Data extraction was performed by two independent reviewers (MO, LG) using a standardized form. To ensure consistency across reviewers, calibration exercises were conducted before starting the review. Disagreements on data extracted were solved through discussion or involving a third reviewer (CM).

### Data items

The following information was extracted from the included studies: bibliographic data (first author, publication year and citation), study characteristics (study design, study period, country, sample size), participant characteristics (gender, age at onset, subtype of GHD), outcome (epidemiological estimates), diagnostic criteria.

### Study risk of bias assessment

Epidemiological studies were assessed by the JBI Critical appraisal checklist for studies reporting prevalence data [8]. The risk of bias assessment was performed by two independent reviewers (MO, LG). Any discrepancies in judgements of risk of bias were resolved by discussion to reach consensus between the two review authors, with a third review author (BP) acting as an arbiter if necessary.

### Statistical analysis

We planned to conduct meta-analyses only in case of homogenous data among the included studies, in terms of study population, outcomes measures, diagnostic criteria, and study design. Otherwise, we decided to describe the studies narratively. Diagnostic accuracy measures have been described as reported in each study or calculated based on available data.

## Results

**Fig. 1:**
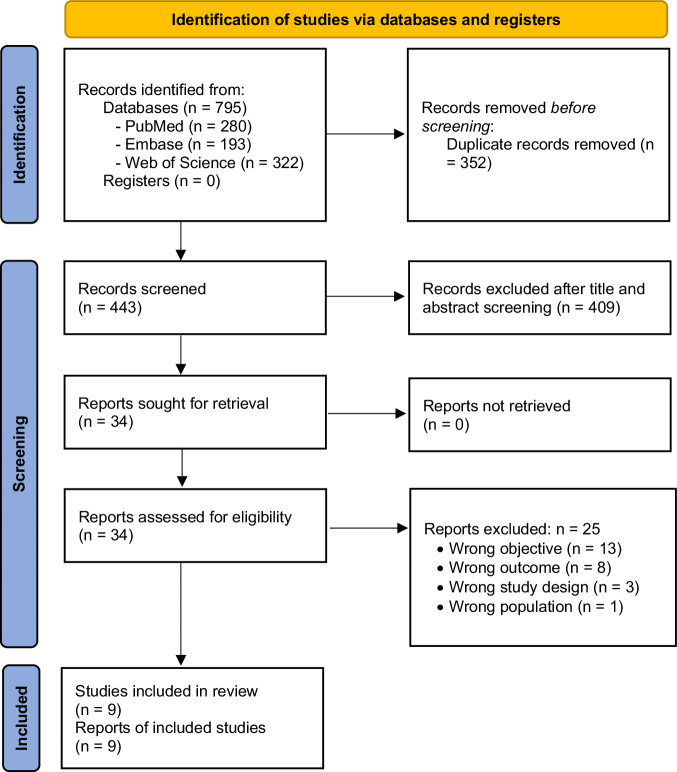
PRISMA 2020 flow diagram (insert here)

We identified 795 records. After duplicates removal, we screened 443 records, from which we reviewed 34 full-text documents, and finally included 9 studies [9–17] (Fig. 1. PRISMA 2020 flow diagram).

Reference lists of relevant articles were also screened, but no extra articles that fulfilled inclusion criteria were found. A list of excluded studies along with reasons for exclusion is provided in Supplementary Table 2.

As anticipated in the methods section, we did not carry out meta-analyses since the included studies were not homogeneous, in terms of etiology of the disease, age difference among included populations, and diagnostic criteria.

We described the main characteristics of the included studies in the Table 1. Among the nine studies included, seven were population-based cohort studies [10–16], and two were population-based cross-sectional studies [9, 17]. Studies were published between 1974 and 2022. Seven studies were conducted in Europe, one in USA and one in China.

**Table 1:** Characteristics of included studies

Study ID	Design	Country	Study period	Population	Disease / Diagnostic criteria	Age at onset	Epidemiological estimates
Bao [9]	Population-based cross-sectional study	China (Beijing)	October 1987-April 1989	6–15 years old elementary and middle school children	Disease: GHD. Criteria: serum GH stimulation tests: GHD confirmed when the peak GH level was < 10 μg/L in two tests	NR	Prevalence: 1/8,646 child with GHD; 1/5777 males and 1/17,253 females
Harju [10]	Population-based retrospective cohort study	Finland	01/01/1998 – 31/12/2017	All children born in Finland registered in Medical Birth Register (0–16 years old)	Disease: GHD + panhypopituitarism. Criteria: two separately performed GH stimulation tests with subnormal responses	7.2 years old boys (median) 8.7 years old girls (median)	Cumulative incidences: 127/100,000 boys, 93/100,000 girls
Lindsay [11]	Population-based cohort study (prevalence prospective study)	USA (Utah)	1990	Kindergarten and elementary school children	Disease: GHD. Criteria: GHD was confirmed if the peak growth hormone level failed to reach 10 ng/ml with two back-to-back provocative tests	NR	Prevalence: 1/3480 child with GHD
Migliaretti [12]	Population-based cohort study (registry study)	Italy (Piedmont)	01/01/2002–31/12/2004	0–18 years old	Disease: hypopituitarism or isolated GHD. Criteria: indication for GH treatment according to the Italian Ministry of Health	NR	Prevalence: 8.62–9.44/10,000; Incidence: 1.86–2.49/10,000
Parkin [13]	Population-based cohort study	UK (Newcastle upon Tyne)	1951–1962	Pediatric patients (age not reported)	Disease: GHD (idiopathic deficiency and deficiency secondary to intracranial disease). Criteria: low serum growth hormone levels after an adequate hypoglycaemic stimulus.	NR	Incidence: 1/30,000 child with GHD
Schweizer [14]	Population-based cohort study	Germany (Baden-Württemberg and Bavaria)	01/01/2000 – 31/12/2001	0–18 years old	Disease: GHD idiopathic, isolated or caused by tumor, encephalitis, and brain anomalies or through a defined genetic defect. Criteria: not reported.	mean (SD): 6.7 (5.1) years	Incidence: total population 3.47 (2.95–4.07)/100,000 Boys: 4.17 (3.37–5.09)/100,000 Girls: 2.75 (2.10–3.54)/100,000
Stochholm [15]	Population-based cohort study	Denmark	1980–1999	0–18 years old	Disease: GHD. Criteria: based on published guidelines with modifications	9.1 years old male (median) 8.6 years old female (median)	Incidence: 2.58/100,000 male children with GHD; 1.70/100,000 female children with GHD
Thomas [16]	Population-based cohort study	Belgium	1986–2001	0–18 years old	Disease: GHD. Criteria: height velocity < the 10th or < the 25th centile for patients with idiopathic and organic GHD respectively, and peak serum GH levels <10 ng/ml during two stimulation tests	IdGHD: 8.6 ± 4.4 (mean) CongGHD: 6.5 ± 4.7 (mean) AcqGHD: 10.9 ± 3.2 (mean)	Prevalence: 18/100,000 (or 1/5600) children with GHD
Vimpani [17]	Population-based cross-sectional study	UK (Edinburgh, Aberdeen, Glasgow)	NR	6–9 years old elementary school children	Disease: severe GHD. Criteria: (a) height over 2.5 standard deviations below mean height for age, (b) height velocity less than 25th centile for chronological age (over 1 year wherever possible), (c) maximum GH concentration of 9 mU/l or less in two or more diagnostic tests.	8.2 ± 0.5 (mean)	Prevalence: 14.5–27/100,000 children with severe GHD

*GHD* growth hormone deficiency, *GH* growth hormone, *IdGHD* idiopathic growth hormone deficiency, *CongGHD* congenital growth hormone deficiency, *AcqGHD* acquired growth hormone deficiency, *NR* not reported

Bao et al. [9] analyzed a cohort of 103,753 elementary and middle school students from 6 to 15 years old in Beijing. The students were measured by school doctors and those with short stature (height <3° percentile) were referred to the hospital for investigation. Of those, 12 children were diagnosed as having GHD (9 males, 3 females). These authors showed that 1/5,777 boys and 1/17,253 girls were affected by GHD, with a prevalence of 1/8,646 in the overall population.

Harju et al. [10] conducted the most recent study between January 1998 and December 2017 in Finland. Using Medical Birth Register (MBR) and the Care Register of Health Care (CRHC) a total of 1,144,503 children (51% boys) were considered. The cumulative incidence (CMI) of 6 disorders responsible for short stature was estimated from birth until the maximum of 16 years of age. At the end of the study period, 790 children and adolescents received a GHD diagnosis (719 isolated GHD, 71 panhypopituitarism). The median age at GHD diagnosis was 8.7 years for girls (range 0–15.3 years) and 7.2 years for boys (range 0–16.0 years). The registered CMI was 127/100,000 for boys and 93/100,000 for girls.

In 1990 Lindsay et al. [11] conducted the first prospective study describing the prevalence of GHD in the USA. More than 140,000 kindergarten and elementary school children were involved and measured. Of those, 17 children were already diagnosed with GHD. The children that were short (height <3rd percentile) and growing less than 5 cm/year were referred to the study physicians for further evaluation. Out of 1,344 short stature children, 16 were newly diagnosed with GHD. The prevalence rate of GHD children was 1/3,480.

Migliaretti et al. [12] extracted data from 918 pediatric patients diagnosed with GHD and treated with rhGH between 2002 and 2004 in Piedmont (Italy) from the GH Register. The epidemiological estimates were calculated considering the Piedmont population each year (619,494 in 2002, 633,420 in 2003–2004). The prevalence rate of children with GHD was 8.62–9.44/10,000 and the incidence rate was 1.86–2.49/10,000.

The first ever recorded study on GHD incidence was conducted by Parkin et al. in 1974 [13] which recorded data of children accepted into the Medical Research Council growth hormone treatment trial in Newcastle Upon Tyne (UK). They showed that the annual incidence of growth hormone deficiency is 1 in 30,000 newborns, given that the number of births in the Newcastle region per year is approximately 48,000.

Schweizer et al. [14] reported an epidemiological study on endocrine disorders conducted in Baden-Württemberg and Bavaria (Germany) in the years 2000–2001. The yearly GHD incidence per 100,000 children at risk was 3.47 (95% CI, 2.95–4.07). The boys showed a higher incidence than girls: 4.17 (95% CI, 3.37–5.09) vs 2.75 (95% CI, 2.10–3.54).

Stochholm et al. [15] used the Cancer Registry, the National Patient Registry and the Case of Death Registry to identify all cases of possible GHD. They found 494 children and adolescents (303 males, 191 females) with GHD in Denmark from 1980 to 1999. The median age at onset was 9.1 years old for boys and 8.6 years old for girls. This study showed that the prevalence of GHD for children and adolescents was respectively 2.58/100,000 for males and 1.70/100,000 for females.

A Belgian study conducted by Thomas et al. [16] evaluated the prevalence and demographic features of childhood GHD in the years 1986–2001. The data was collected from the Belgian Study Group for Pediatric Endocrinology (BSGPE), a committee composed of medical experts that selected patient candidate for rhGH treatment. The prevalence estimates were calculated on the basis of the Belgian demographic data provided by the National Institute of Statistics (2.2 million children and adolescents <18 years old) and 740 patients selected by BSGPE: the resulting prevalence was 18/100,000 (or 1/5,600) children with GHD. The mean age at onset was 8.6 ± 4.4 for children with idiopathic GHD, 6.5 ± 4.7 for children with congenital GHD and 10.9 ± 3.2 for children and adolescents with acquired GHD.

In 1977 Vimpani et al. [17] wrote about the Scottish Survey for Short Stature that screened 48,221 elementary school children from Edinburgh, Aberdeen, and Glasgow. After a first selection that measured height, 280 short stature children (height < 2.5 standard deviation) were screened for GHD. Nine children were newly diagnosed with GHD while 4 already had a GHD diagnosis previously to the survey. The estimated prevalence of severe GHD (see diagnostic criteria reported in Table 1) in elementary school children was about 14.5–27/100,000.

### Risk of bias in included studies

The methodological quality of the included studies was assessed using the JBI (Joanna Briggs Institute) critical appraisal checklist for prevalence studies (Supplementary Table 3).

One study [16] fulfilled 8 out of 9 items, with one item in the checklist not applicable (adequacy of response rate). Seven studies [9–14, 17] had inappropriate samples to address the target population. Four studies screened kindergarten, elementary or middle school children and adolescents, missing all the children between 0–6 years old or older than 9–16 years old. Three other studies included also other forms of GHD or other diseases [12–14].

In most studies, the measuring conditions were unclear (item n. 7 on the checklist), except for Thomas et al. [16]. Out of 5 studies [10–12, 14, 15] that had 6/9 items satisfied, three of them had one item not applicable (adequacy of response rate). The study that scored the least items on the checklist was the oldest study by Parkin [13], due to inadequate sample size, inappropriate statistical analysis, and unclear response rate.

## Discussion

To our knowledge, this is the first epidemiological systematic review on pediatric GHD, therefore it was not possible to compare our results to others. Most of the included studies were European, with only two exceptions, one study from USA and another from China. Half of the studies were published before the year 2000. The study periods ranged from 1951 to 2017. A possible reason why there are not many studies regarding GHD epidemiology and mostly located in Europe could be the lack of population-based registries worldwide. Another reason could be the lack of public funding for research, especially in countries without a public health system.

Included studies reported a range of prevalence of 1/1107–1/8,646. A study based on a registry of GH users in Piedmont region (Italy) [12] reported the highest mean prevalence (1/1107). In the included studies, the mean incidence ranged from 1/28,800 to 1/46,700 cases per year. Harju reported a 20-year cumulative incidence of 127/100,000 for boys and 93/100,000 for girls.

As for populations, 4 studies included children with isolated GHD, and one study reported prevalence for severe GHD. Four studies included mixed populations: Harju et al. [10] considered together children with panhypopituitarism or isolated GHD; Migliaretti et al. [12] included children with hypopituitarism or isolated GHD; Parkin [13] and Schweizer et al. [9] reported on children with idiopathic GHD or secondary to other causes.

Not all studies considered the entire pediatric population (0–18): Bao et al. [9] considered 6–15 years old children, Harju et al. [10] 0–16 years old children, Lindsay et al. [11] kindergarten and elementary school children, Parkin [13] pediatric patients (age not reported), and Vimpani et al. [17] 6–9 years old elementary school children. Considering the role of puberty on the growth pattern and the physiological variability in the growth acceleration during puberty, to consider all age periods is crucial for a comprehensive approach.

GH stimulation tests are the gold standard to diagnose GHD, however there is still no standardized approach to the GHD diagnosis. This problem is reflected by the variability of criteria applied in previously analyzed epidemiologic studies. Regarding diagnostic criteria, most studies confirmed a GHD diagnosis based on two separately performed GH stimulation tests. In some studies, the cut-off indicating GHD was a serum level of GH < 10 ng/ml. Three studies did not report any cut-off. In the study of Vimpani et al. [17] the initial screening for GHD was by an insulin hypoglycemic test, an extended glucose tolerance test or exercise. Additionally, variables including test length, growth hormone assay and diagnostic cut off affect results. A more uniform diagnostic approach worldwide for GHD is needed. For example, adequacy of a peak GH concentration below 10 ng/ml after GH stimulation test for the diagnosis was already discussed in the 2000 consensus of the GH Research Society [2], suggesting to revise the value using newer monoclonal-based assays and recombinant hGH reference preparations. In the 2019 audit by Binder et al. [18] has been reported that 7 out of 9 countries abandoned the traditional cutoff of GH < 10 ng/ml, and most European countries ranged from 6 to 8 ng/ml. In addition, several provocative agents are used to test GH, such as arginine, clonidine, glucagon, insulin, and L-dopa; different combinations are used by different countries.

As for priming with sex hormones prior to GH stimulation test, Grimberg et al. [5] suggested it as a conditional recommendation, while Binder et al. [18] reported this practice as a matter of debate, due to a better specificity of GH tests but a loss of physiological response. Therefore, considering all the existing diagnostic variabilities, new international evidence-based guidelines are required.

As for the methodological quality of included studies, all but one study [13] satisfied the majority of the checklist items. The diagnosis of GHD remains difficult and often delayed, and literature data highlighted the unsatisfactory nature of our current diagnostic process.

Considering that included studies are mostly European, the provided estimates cannot be considered global and furthermore it is necessary to conduct new studies comprehensive of countries and populations not included in this review. To our knowledge, there have been only a few nationwide studies using uniform diagnostic and classification criteria for all citizens.

International multicentre studies are needed to compare epidemiological estimates of GHD among different ethnic groups and at different ages. The scientific community should move forward to improve the diagnosis of GHD and to harmonize protocols for diagnosis and management of GHD between countries and regions. Since the only available treatment for GHD is human recombinant GH, and its considerable cost to the national healthcare systems, it would be crucial to know the accurate prevalence of GHD in every country to promote an appropriate resource allocation.

### Supplementary information

Supplementary Table 1

Supplementary Table 2

Supplementary Table 3

## Data Availability

All relevant data are within the manuscript and its Supporting Information files.
