# Supplementary material for: Epidemiology of growth hormone deficiency in children and adolescents: a systematic review
Source: Endocrine. 2024 Mar 18;85(1):91–8. doi: 10.1007/s12020-024-03778-4 (PMC11246253; doi:10.1007/s12020-024-03778-4)
Supplement: Supplementary file 1 — Supplementary Table 1 [file 12020_2024_3778_MOESM1_ESM.docx]

**Supplementary Table 1. Literature search strategy**

|  | **PubMed, 30/07/2023** |  |
| --- | --- | --- |
| Search | Query | Results |
| #1 | growth hormone deficiency OR GHD | 18,776 |
| #2 | epidemiology[Title] OR prevalence[Title] OR incidence[Title] OR burden[Title] OR population-based[Title/Abstract] | 537,323 |
| #3 | #1 AND #2 | **280** |

|  | **Embase, 30/07/2023** |  |
| --- | --- | --- |
| Search | Query | Results |
| #1 | ‘growth hormone deficiency’ OR GHD | 17,171 |
| #2 | epidemiology:ti OR prevalence:ti OR incidence:ti OR burden:ti OR population-base:ti,ab | 530,739 |
| #3 | #1 AND #2 AND ([article/lim OR [article in press/lim OR [review/lim]) | **193** |

|  | **Web of Science, 30/07/2023** |  |
| --- | --- | --- |
| Search | Query | Results |
| #1 | TS=(growth hormone deficiency OR GHD) | 17,709 |
| #2 | (TI=(epidemiology OR prevalence OR incidence OR burden) OR TS=(population-based)) | 654,011 |
| #3 | #1 AND #2 | 353 |
| #4 | #1 AND #2 and Article or Review Articles (Document Types) | **322** |
