# Supplementary material for: Epidemiology of growth hormone deficiency in children and adolescents: a systematic review
Source: Endocrine. 2024 Mar 18;85(1):91–8. doi: 10.1007/s12020-024-03778-4 (PMC11246253; doi:10.1007/s12020-024-03778-4)
Supplement: Supplementary file 2 — Supplementary Table 2 [file 12020_2024_3778_MOESM2_ESM.docx]

**Supplementary Table 2. List of excluded studies and reasons for exclusion.**

| **Reference** | **Reason for exclusion** |
| --- | --- |
| 1. Al-Agha, A. E., et al. (2011). Prevalence of growth hormone deficiency among short children at King Abdul-Aziz university hospital: A 5-year experience. Pakistan Paediatric Journal 35(2): 90-93. | wrong outcome |
| 1. Arnao, M. D. R., et al. (2014). The DATAC study: A new growth database. Description of the epidemiology, diagnosis and therapeutic attitude in a group of Spanish children with short stature. Journal of Pediatric Endocrinology and Metabolism 27(11-12): 1201-1208. | wrong outcome |
| 1. Audi, L., et al. (2002). Long-term GH therapy: Epidemiology and auxologic outcome. Horm Res 57(3-4): 113-119. | wrong study objective |
| 1. Bagnasco, F., et al. (2017). PREVALENCE AND CORRELATES OF ADHERENCE IN CHILDREN AND ADOLESCENTS TREATED WITH GROWTH HORMONE: A MULTICENTER ITALIAN STUDY. Endocrine Practice 23(8): 929-941. | wrong study objective |
| 1. Berglund, A., et al. (2015). Growth hormone replacement does not increase mortality in patients with childhood-onset growth hormone deficiency. Clin Endocrinol (Oxf) 83(5): 677-683. | wrong study objective |
| 1. Brod, M., et al. (2017). Understanding burden of illness for child growth hormone deficiency. Quality of Life Research 26(7): 1673-1686. | wrong study objective |
| 1. Brod, M., et al. (2017). Understanding Treatment Burden for Children Treated for Growth Hormone Deficiency. Patient-Patient Centered Outcomes Research 10(5): 653-666. | wrong study objective |
| 1. Carel, J. C., et al. (2012). Long-term mortality after recombinant growth hormone treatment for isolated growth hormone deficiency or childhood short stature: preliminary report of the French SAGhE study. J Clin Endocrinol Metab 97(2): 416-425. | wrong outcome |
| 1. Fideleff, H. L., et al. (2016). Burden of Growth Hormone Deficiency and Excess in Children. Growth Hormone in Health and Disease. F. F. Casanueva. 138: 143-166. | wrong study design |
| 1. Giovenale, D., et al. (2006). The prevalence of growth hormone deficiency and celiac disease in short children. Clin Med Res 4(3): 180-183. | wrong outcome |
| 1. Gjikopulli, A., et al. (2015). Final Height in Children with Idiopathic Growth Hormone Deficiency treated with Growth Hormone: Albanian experience. Curr Health Sci J 41(1): 22-28. | wrong study objective |
| 1. Golden, S. H., et al. (2009). Prevalence and incidence of endocrine and metabolic disorders in the united states: A comprehensive review. Journal of Clinical Endocrinology and Metabolism 94(6): 1853-1878. | wrong study design |
| 1. Grimberg, A. and G. P. Kanter (2019). US Growth Hormone Use in the Idiopathic Short Stature Era: Trends in Insurer Payments and Patient Financial Burden. Journal of the Endocrine Society 3(11): 2023-2031. | wrong study objective |
| 1. Jakobsen, L. K., et al. (2023). Diagnosis and Incidence of Congenital Combined Pituitary Hormone Deficiency in Denmark-A National Observational Study. Journal of Clinical Endocrinology & Metabolism. | wrong population |
| 1. Kaplowitz, P., et al. (2021). Economic burden of growth hormone deficiency in a US pediatric population. J Manag Care Spec Pharm 27(8): 1118-1128. | wrong study objective |
| 1. Kremidas, D., et al. (2013). Administration burden associated with recombinant human growth hormone treatment: perspectives of patients and caregivers. J Pediatr Nurs 28(1): 55-63. | wrong study objective |
| 1. Loftus, J., et al. (2021). Suboptimal adherence to daily growth hormone in a US real-world study: an unmet need in the treatment of pediatric growth hormone deficiency. Curr Med Res Opin 37(12): 2141-2150. | wrong study objective |
| 1. Orso, M., et al. (2022). Pediatric growth hormone treatment in Italy: A systematic review of epidemiology, quality of life, treatment adherence, and economic impact. PLoS One 17(2): e0264403. | wrong study design |
| 1. Puga González, B., et al. (2010). The effects of growth hormone deficiency and growth hormone replacement therapy on intellectual ability, personality and adjustment in children. Pediatr Endocrinol Rev 7(4): 328-338. | wrong study objective |
| 1. Quitmann, J., et al. (2019). First-year predictors of health-related quality of life changes in short-statured children treated with human growth hormone. J Endocrinol Invest 42(9): 1067-1076. | wrong study objective |
| 1. Rodríguez Arnao, M. D., et al. (2014). The DATAC study: a new growth database. Description of the epidemiology, diagnosis and therapeutic attitude in a group of Spanish children with short stature. J Pediatr Endocrinol Metab 27(11-12): 1201-1208. | wrong outcome |
| 1. Smyczynska, J., et al. (2014). Incidence and predictors of persistent growth hormone deficiency (GHD) in patients with isolated, childhood-onset GHD. Endokrynol Pol 65(5): 334-341. | wrong outcome |
| 1. Sommer, R., et al. (2015). Understanding the impact of statural height on health-related quality of life in German adolescents: a population-based analysis. European Journal of Pediatrics 174(7): 875-882. | wrong study objective |
| 1. Velayutham, K., et al. (2017). Prevalence and Etiological Profile of Short Stature among School Children in a South Indian Population. Indian J Endocrinol Metab 21(6): 820-822. | wrong outcome |
| 1. Zayed, A. A., et al. (2014). The prevalence of isolated growth hormone deficiency among children of short stature in Jordan and its relationship with consanguinity. Clin Endocrinol (Oxf) 81(6): 876-882. | wrong outcome |
