# Supplementary material for: Epidemiology of growth hormone deficiency in children and adolescents: a systematic review
Source: Endocrine. 2024 Mar 18;85(1):91–8. doi: 10.1007/s12020-024-03778-4 (PMC11246253; doi:10.1007/s12020-024-03778-4)
Supplement: Supplementary file 3 — Supplementary Table 3 [file 12020_2024_3778_MOESM3_ESM.docx]

**Supplementary Table 3**

**Risk of bias assessment**

|  | Bao 1992 | Harju 2022 | Lindsay 1994 | Migliaretti 2006 | Parkin 1974 | Schweizer 2010 | Stochholm 2006 | Thomas 2004 | Vimpani 1977 |
| --- | --- | --- | --- | --- | --- | --- | --- | --- | --- |
| 1. Was the sample frame appropriate to address the target population? | No | No | No | No | No | No | Yes | Yes | No |
| 2. Were study participants sampled in an appropriate way? | Yes | Yes | Yes | Yes | Yes | Yes | Yes | Yes | Yes |
| 3. Was the sample size adequate? | Yes | Yes | Yes | Yes | No | Yes | Yes | Yes | Yes |
| 4. Were the study subjects and the setting described in detail? | Yes | Yes | Yes | Yes | Yes | Yes | Yes | Yes | Yes |
| 5. Was the data analysis conducted with sufficient coverage of the identified sample? | Unclear | Yes | Yes | Yes | Yes | Yes | Yes | Yes | Unclear |
| 6. Were valid methods used for the identification of the condition? | Yes | Yes | Yes | Yes | Yes | Yes | Yes | Yes | Yes |
| 7. Was the condition measured in a standard, reliable way for all participants? | Unclear | Unclear | Unclear | Unclear | Unclear | Unclear | Unclear | Yes | Unclear |
| 8. Was there appropriate statistical analysis? | Yes | Yes | Yes | Yes | No | Yes | Yes | Yes | Yes |
| 9. Was the response rate adequate, and if not, was the low response rate managed appropriately? | Yes | Not applicable | Yes | Not applicable | Unclear | Not applicable | Not applicable | Not applicable | Yes |
